# The *Journal of Orthopaedics and Traumatology* in the 15th year from foundation: actual achievements and future directions

**DOI:** 10.1007/s10195-014-0326-7

**Published:** 2014-11-18

**Authors:** Luca Pierannunzii, Marco d’Imporzano

**Affiliations:** 1 Istituto Ortopedico Gaetano Pini, Piazza A. Ferrari, 1, 20122 Milan, Italy; 2 Istituto Auxologico Italiano, Via Mercalli, 28, 20122 Milan, Italy

In 2000 Francesco Pinino founded the *Journal of Orthopaedics and Traumatology* (JORT), with the plain intention of providing the Italian Society of Orthopaedics and Traumatology (SIOT) with a powerful communication tool, through which Italian authors could spread the results of their research activity beyond the borders of Italian language, and contemporarily the contributions of foreign scientists could be published in an Italian-based journal with a clear propensity for a worldwide circulation.

At the beginning the former effect was more evident, mostly because the journal still needed to be acknowledged by the international community, but in recent years the latter became largely prevalent and made JORT a globally recognized scientific journal dealing with all aspects of musculoskeletal medicine and surgery. The submissions analysis confirms this development: in 2010, 191 manuscripts were submitted, 68.6 % of which had foreign authorship, while in 2013, 223 manuscripts were received, and 81.6 % of them had a foreign corresponding author. This positive trend seems to be confirmed in 2014, with further rise of submissions and a remarkable increase of contributions originating from countries with top quality scientific production.

## Indexing

Pubmed/MEDLINE, Scopus, EMBASE, Scimago, EBSCO, and Google Scholar are only a few of the abstracting and indexing institutes, databases, and search engines in which JORT is listed. In 2008 the inclusion in Pubmed strongly improved the visibility of our journal and represented a milestone in JORT’s history. The subsequent year it was indexed in MEDLINE by the National Library of Medicine (NLM), thus receiving an important external validation both of the scientific quality and of the editorial production.

## Citations

The number of citations that an article receives, with all its derived statistics, is likely the most effective estimate of the impact the article is having on the scientific community. For these reasons, the same statistics have been extrapolated to describe firstly authors’ performance and subsequently journals’ performance.

Scimago is a source of important information about journals’ citations, providing statistical analyses based on the Scopus database. Unfortunately, 2014 results are still unavailable at the present time, but 2013 data confirm that JORT ranks for the third consecutive year in the first quartile both in medicine and in surgery, and in the second quartile in orthopedics and sports medicine. The H-index is as high as 15, meaning that 15 published manuscripts received at least 15 citations. The “cites per document (2 years)”, i.e., the average number of citations that articles published in years *x* − 1 and *x* − 2 receive in year *x*, doubled in 3 years, jumping from 0.82 in 2010 to 1.68 in 2013 [1].

In Google Scholar, JORT displayed a similar remarkable progress, with an H5-index as high as 19, meaning that 19 articles published from 2009 to 2013 received at least 19 citations [2]. It was 17 the year before.

The *Journal of Orthopaedics and Traumatology* is not yet indexed in the Journal Citation Reports^®^ (JCR), thus we cannot provide an impact factor (IF). Although most “non-impacted” journals offer their authors and readers unofficial or virtual IF, such calculations are somehow speculative, and often their publication on the journal webpage represents a bold move. This is because the difference between the virtual and official IF, calculated by ISI Web of Science in cases of inclusion, may be relevant, due to different evaluations of the citable items, to computation of multiple citations from the same article, etc. Under these premises and with all due caution, our editorial office attempted to calculate the citable items published in 2011 and 2012 and their citations collected from publications listed in JCR and from the same journal (self-citations) in 2013. The ratio between citations (91) and citable items (72) turned out to be as high as 1.264. This favorable 2013 ratio confirmed and slightly improved what was obtained in 2012 (1.259), and a similar result is expected in 2014, although relevant yearly fluctuations are possible for small-size journals like JORT.

## Open access

The *Journal of Orthopaedics and Traumatology* has been a full open access journal since 2008. This means that all the contents are freely available to readers without any subscriptions. This choice strongly contributed to facilitate JORT’s usage, achieving very high download rates: in 2012, 34,734 full-text articles were downloaded; last year the count rose up to 83,669, and preliminary 2014 data confirm this trend. This helped noticeably the citability of the published papers.

The JORT’s full open access policy shows the peculiarity that no fees are asked from authors, since the whole publication cost is waived by the Italian Society of Orthopaedics and Traumatology (SIOT). This policy, followed from the very beginning, aims at guaranteeing all the authors, Italian and foreign, equal rights to publish their results regardless of their economic resources. At the same time, peer-review transparency could not be questioned, as it occurred elsewhere for systems where authors pay to publish through article processing charges. In society’s purposes, the no-fee policy would have led to the most unconditional results sharing, with very little chance that economical filters might influence the publication of important findings both positively or negatively. Editors, authors, and readers cannot but thank SIOT for such an important and burdensome commitment.

## Manuscripts processing and editorial board renewal

In 2014 JORT’s editorial board was renovated in order to deal with a higher submissions volume without compromising duration and quality of the editorial processing. Our journal keeps adhering to a double-blind, double peer-review process, which means that all the papers positively screened by the executive editor undergo a double-blind evaluation by two independent reviewers, selected within the panel of Scientific Referees [3] for their expertise in the article field. The subordinate editor who is in charge of the manuscript receives the reviewers’ reports and conveys their own judgement to the editor-in-chief, who takes the final decision (rejection, acceptance, or revision). Third reviewers are often involved when the former two provide contrasting suggestions.

This year, the Consulting Editors Board, which was the board of subordinate editors supervising the peer-review process, changed its name to Associate Editors Board, with an increased number of members and, more importantly, a declared and limited area of expertise for each member. The title change is far more substantial than formal, since the associate editor is not just an editor-in-chief’s consultant, but a key figure of the editorial workflow, whose activity is strictly and transparently related to his special expertise. The choice of the new members depended both on their outstanding scientific activity and on the past contribution to the journal as efficient peer reviewers or proficient advisors, while former consulting editors were confirmed as associate editors based on their performance.

Next to the former confirmed editors, the *Journal of Orthopaedics and Traumatology* welcomes the new associate editors Matthew P. Abdel (Mayo Clinic, Rochester, NY, USA), Max Aebi (Salem-Hirslanden Hospital, Bern, Switzerland), Annunziato Amendola (University of Iowa, Iowa City, IA, USA), Michael Cross (Hospital for Special Surgery, NY, USA), Max Haerle (OKM, Markgröningen, Germany), Massimo Innocenti (Azienda Ospedaliero Universitaria Careggi, FI, Italy), Cyril Mauffrey (Denver Health Medical Center, Denver, CO, USA), Pietro Randelli (Policlinico San Donato, Milano, Italy), and John Sperling (Mayo Clinic, Rochester, NY, USA).

## Production and post-production data

Notwithstanding the growing trend of submissions, JORT is still a small-volume journal, with four issues and about 40 published articles per year. This volume is the consequence of a strict manuscript selection obtained through scrupulous peer-review. The rejection rate, i.e., the fraction of submissions that are lastly not accepted for publication, increased progressively from 60 % in 2009 to beyond 80 % in 2012 and stabilized at this level. In the light of our recent submissions’ quality, this rejection rate guarantees an adequate scientific level of the accepted manuscripts.

On a regular basis the publisher provides the editorial board with data about “usage” and citation of the journal contents. This information is exploited by the editor-in-chief to plan the editorial policy. Rarely is such information shared with readers, although it might be interesting and possibly beneficial for most potential authors.

Remarkably, the two most read papers in the last 3 years were an original article dealing with limb reconstruction after tumor resection [4] and a review article about patellar tendinopathy treatment [5]; both were downloaded more than 2,000 times since their online publication occurred, respectively, in February 2013 and December 2012.

Interestingly, within the sources of citations listed by ISI Web of Science, the knee seems to be the most citable subject of papers published in 2012–2013, since the three (equally) most cited papers were two review articles [5, 6] and an original article [7] about this joint. The same search performed in the previous biennium (2010–2011) led to quite different results, the three most cited papers exclusively or mainly dealing with the hip joint [8–10].

In Scopus, the most cited article of the period 2012–2013 reports an in vitro study about a novel technique for the treatment of depressed articular fractures [11]. This result conforms with the recent increase of basic and translational research papers among our submissions, inducing the editor-in-chief to nominate a dedicated associate editor to manage these contributions. More pre-clinical research papers are expected to be published in future issues, and we strongly believe this phenomenon will positively influence originality and quality of the clinical research papers too, meanwhile conveying new stimuli to the orthopedic community hitherto less familiar with this kind of reading.

## Future directions

The editorial policy of medical journals is influenced by the purpose of sustaining and increasing the journal’s citability. Although citation-based scientometrics are often overestimated, they still represent the most common and validated way to evaluate the scientific quality of a journal's production. In medical literature, review articles are usually highly cited papers and case reports the least cited ones, and this has led many journals to offer more and more invited reviews and exclude case reports from possible contributions. The JORT’s editorial board decided not to influence or filter the authors’ production, promoting spontaneous submission and guaranteeing proper consideration for all the manuscript types. So far the citations analysis confirms the efficacy of such a liberal editorial policy. The current issue building is substantially representative of our submissions, with original articles prevailing over the other types of manuscripts, as it is supposed to be for every healthy and vital scientific journal. All the fields of clinical and pre-clinical research are welcome, as long as our ethical standards are fulfilled. Case reports are not excluded nor will be, since they have a definite role in medical literature as potential triggers for future research. Obviously, strict conformity to the rigorous eligibility criteria is requested [12], neglecting which leads to prompt editorial rejection. Letters to the editor about recently published papers are encouraged when pertinent and stimulating, since peer-review does not end with publication, and the constructive debate between authors and readers is the life blood of every journal.

As for the future, the *Journal of Orthopaedics and Traumatology* aims at consolidating its position of serious and reliable scientific journal dealing with all the aspects of musculoskeletal medicine and surgery, offering quality selection through rigorous and transparent peer-review, clear visibility through full open access policy, and high citability by means of effective indexing in most databases and search engines. We all—editors, authors, and readers—are indebted to the founder Prof. Francesco Pipino, who recently passed away, for leaving such an impressive inheritance to the Italian orthopedic community, and cannot but feel a strong commitment to the continuation of his project.
